# Shukla-Vernon Syndrome: A Second Family with a Novel Variant in the *BCORL1* Gene

**DOI:** 10.3390/genes12030452

**Published:** 2021-03-22

**Authors:** Babylakshmi Muthusamy, Anikha Bellad, Satish Chandra Girimaji, Akhilesh Pandey

**Affiliations:** 1 Institute of Bioinformatics, International Technology Park, Bangalore 560066, India; anikha@ibioinformatics.org; 2 Manipal Academy of Higher Education, Manipal 576104, India; 3 Center for Molecular Medicine, National Institute of Mental Health and Neurosciences (NIMHANS), Hosur Road, Bangalore 560029, India; 4 Department of Child and Adolescent Psychiatry, NIMHANS, Hosur Road, Bangalore 560029, India; 5 Department of Laboratory Medicine and Pathology, Mayo Clinic, Rochester, MN 55905, USA; 6 Center for Individualized Medicine, Mayo Clinic, Rochester, MN 55905, USA

**Keywords:** X-linked, intellectual disability, transcription repression, BCOR, BCL-6, co-repressor

## Abstract

Shukla-Vernon syndrome (SHUVER) is an extremely rare neurodevelopmental disorder characterized by global developmental delay, intellectual disability, behavioral anomalies, and dysmorphic features. Pathogenic variants in the *BCORL1* gene have been identified as the molecular cause for this disorder. The *BCORL1* gene encodes for BCL-6 corepressor-like protein 1, a transcriptional corepressor that is an integral component of protein complexes involved in transcription repression. In this study, we report an Indian family with two male siblings with features of Shukla-Vernon syndrome. The patients exhibited global developmental delay, intellectual disability, kyphosis, seizures, and dysmorphic features including bushy prominent eyebrows with synophrys, sharp beaked prominent nose, protuberant lower jaw, squint, and hypoplastic ears with fused ear lobes. No behavioral abnormalities were observed. Whole exome sequencing revealed a novel potentially pathogenic arginine to cysteine substitution (p.Arg1265Cys) in the BCORL1 protein. This is the second report of Shukla-Vernon syndrome with a novel missense variant in the *BCORL1* gene. Our study confirms and expands the phenotypes and genotypes described previously for this syndrome and should aid in diagnosis and genetic counselling of patients and their families.

## 1. Introduction

Shukla-Vernon syndrome (SHUVER; OMIM: #301029) is a rare X-linked recessive neurodevelopmental disorder originally described in two unrelated patients from the United States and three affected siblings in an Indian family [1]. Patients with Shukla-Vernon syndrome exhibit global developmental delay, intellectual disability, behavioral difficulties including aggressive behavior, ADHD, autism spectrum disorder, stereotypic hand movements, drooling of saliva, and dysmorphic features including dolichocephaly, tall forehead, broad base of nose, thick vermilions, dysmorphic ears and hypertelorism/strabismus, down slanting palpebral fissures, and reduced muscle tone [1]. The degree of intellectual disability varies from mild to severe among these patients. In addition, cerebellar atrophy was observed in brain imaging and some patients had epilepsy [1]. Pathogenic variants in the *BCORL1* gene have been identified as the molecular cause for the Shukla-Vernon syndrome. Carrier females may have mild manifestations [1]. Prior to description of this syndrome, a hemizygous missense variant p.Asn820Ser in BCORL1 was reported in a non-consanguineous family comprising of two males exhibiting clinical phenotypic features of severe intellectual disability, coarse face, and hypotonia [2].

The *BCORL1* gene encodes a transcription co-repressor that was identified as a homolog of BCL6 corepressor (BCOR), a corepressor for transcription repressor *BCL6*. However, BCORL1 does not associate with BCL6 and is believed to have a distinct role in human cells [3]. BCORL1 acts as a corepressor by being an integral component of three transcription regulatory complexes by interacting with (1) class II histone acetyltransferases and deacetylases (HDACs), HDAC4, HDAC5, and HDAC7 [3], (2) C-terminal binding proteins (CtBP) [3], and (3) polycomb group ring finger 1 (PCGF1) and polycomb group ring finger 3 (PCGF3) proteins, components of the polycomb repression complex 1 (PRC1.1), which is essential for histone methylation for gene silencing [4,5]. However, the exact mechanisms through which *BCORL1* causes intellectual disability and associated phenotypes are not yet determined.

Here, we describe a second Indian family with two affected males exhibiting clinical features of intellectual disability, global developmental delay, and dysmorphism. Whole exome sequencing revealed a novel, potentially pathogenic, missense variant in *BCORL1* that segregated in the family with X-linked recessive inheritance.

## 2. Materials and Methods

### 2.1. Patients

**Figure 1:**
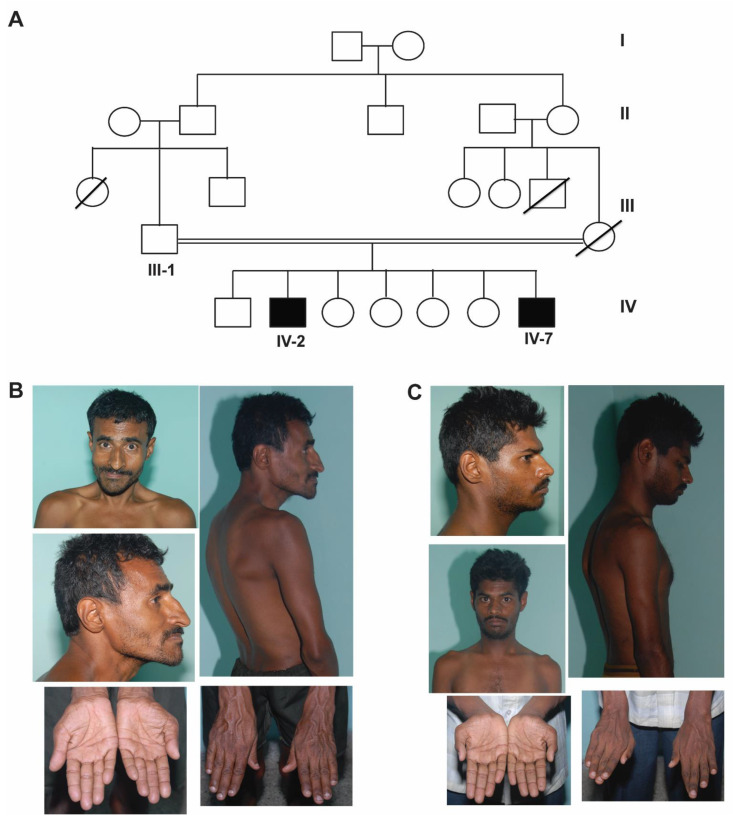
Pedigree and clinical features. (**A**) pedigree, (**B**) clinical photographs of the older affected sibling (IV-2) showing dysmorphism including broad forehead, synophrys, sharp beaked nose, protuberant lower jaw, squint and hypoplastic ears with fused ear lobes, hand anomalies including transverse palmar crease, long fingers, and clinodactyly and kyphoscoliosis, and (**C**) clinical photographs of the younger affected sibling (IV-7) showing dysmorphism including low hairline, bushy eyebrows with synophrys, prominent beaked nose, hypoplastic fused ear lobes, hand anomalies including long fingers, clinodactyly, wasting of thenar and hypothenar eminence, overlapping second finger on pronated hand, and kyphosis.

Two male siblings born of third-degree consanguineous marriage (cousin marriage) of Indian origin presented to the psychiatry outpatient clinic at NIMHANS, Bangalore where a detailed clinical workup was performed. Their clinical features are tabulated in Table 1 and a four-generation pedigree is depicted in Figure 1A. The patients were evaluated by a psychiatrist at the National Institute of Mental Health and Neurosciences (NIMHANS) based on International Classification of Diseases- Tenth revision (ICD-10) criteria. This study was approved by the ethics committee of NIMHANS, Bangalore. The patients lacked mental capacity, due to which, written informed consent for the patients to participate in this study and for their clinical details and clinical photographs to be published was obtained from the father.

Patient 1 (IV-2) was a 35-year-old male who was born by an uncomplicated vaginal delivery and had an uneventful perinatal period. He had history of significant global developmental delay with marked motor and speech delay. He also had a history of three episodes of deviation of the angle of mouth to the right side with inability to close the left eye with no weakness of the opposite side of the body suggestive of left-sided lower motor neuron facial palsy at the age of 10, 13, and 17 years. This was probably because of recurrent Bell’s palsy. The facial weakness was not evident at the time of examination. Physical examination revealed kyphoscoliosis, dysmorphic features like broad forehead, bushy prominent eyebrows with synophrys, sharp beaked prominent nose, protuberant lower jaw, squint and hypoplastic ears with fused ear lobes along with transverse palmar crease, long fingers, and clinodactyly. Neuropsychiatric examination revealed an IQ of 39, indicating a moderate degree of intellectual disability, and brisk deep tendon reflexes. The rest of the systemic examination was unremarkable (Figure 1B and Table 1).

Patient 2 (IV-7) was a 24-year-old male who was also born by an uncomplicated vaginal delivery and had an uneventful perinatal period. He presented with intellectual disability and had a history of global developmental delay. He also had a history of uncontrolled generalized tonic–clonic seizures since the age of 7 years and was on anti-epileptics. Physical examination revealed kyphosis, dysmorphic features including low hairline, bushy eyebrows with synophrys, prominent beaked nose, hypoplastic fused ear lobes, long fingers, clinodactyly, wasting of thenar and hypothenar eminence, and overlapping second finger on pronated hand. Neuropsychiatric examination revealed an IQ of 47, indicating moderate degree of intellectual disability, and brisk deep tendon reflexes. The rest of systemic examination was unremarkable (Figure 1C and Table 1).

### 2.2. G-Banded Karyotyping and Whole Exome Sequencing

Blood samples were collected from both affected siblings and the unaffected father. DNA was isolated from the blood samples using QIAamp DNA minikit (Qiagen, Germantown, MD, USA) as per the manufacturer’s protocol. In order to identify chromosomal abnormalities, we performed G-banded karyotyping in both affected siblings. We carried out whole exome sequencing on both affected patients and the unaffected father. Karyotyping, whole exome sequencing and data analysis were performed as described previously (Muthusamy, Selvan et al. 2017). Briefly, DNA library preparation was carried out using SureSelectXT Human All Exon V5+UTR kit and the captured library was sequenced on Illumina HiSeq X10 platform to generate 2 × 150 bp sequence reads to achieve a targeted read depth of coverage of about 100×. The quality filtered raw reads were aligned to the human reference genome (hg19) using Genome Analysis Toolkit (GATK) best practices workflow [6]. Joint variant calling was carried out using the sequencing data obtained from the two affected individuals and the unaffected father. Variants with minor allele frequency < 0.01 were retained after comparing with the 1000 genomes project [7] and gnomAD databases (https://gnomad.broadinstitute.org/, accessed on 15 February 2021). Exonic and splice signal variants were retained and synonymous variants were removed. Autosomal and X-linked recessive inheritance pattern were applied to identify the potentially pathogenic variants. The functional impact of the shortlisted variants was assessed using Sorting Intolerant From Tolerant (SIFT) [8], Polyphen-2 [9], MutationTaster [10] and CADD [11] software tools. The pathogenicity of the variants was analyzed using extensive literature curation.

Homozygosity mapping was carried out using AutEx algorithm of FILTUS software [12]. Parents’ relation was chosen as 1st–2nd cousins and the posterior threshold was set as 0.5 for the minimum segment size of 1 Mb with at least 100 variants. The resulted autozygosity regions were manually analyzed and visualized using integrated genomics viewer (IGV).

In order to validate the identified pathogenic *BCORL1* variant, we performed PCR amplification and Sanger sequencing using a forward primer: 5′-CCCGAACGGTACAGCTAATAA-3′ and a reverse primer: 5′-CTTCTTGCTCTGTCAGGTACTC-3′. The identified *BCORL1* variant was visualized for the pathogenic variant in the patients and unaffected father.

## 3. Results

**Figure 2:**
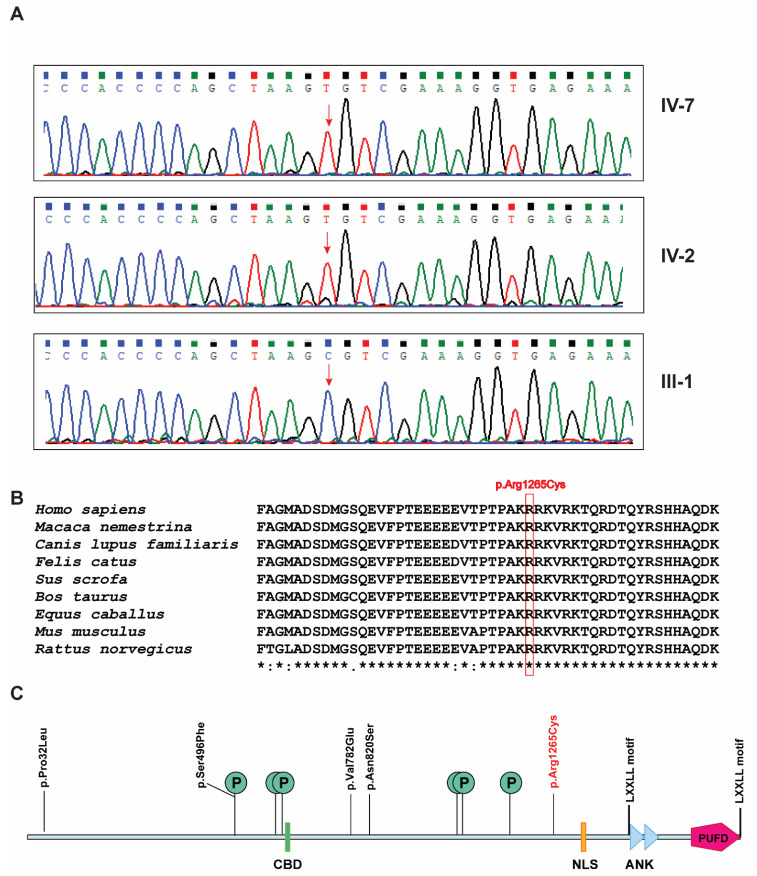
BCORL1 pathogenic variant. (**A**) Sanger sequencing results of *BCORL1* gene variant g.129159069C>T in two affected individuals (hemizygous) and the unaffected father (heterozygous). The site of the pathogenic variant is marked by a red arrow. (**B**) Conservation of the region with the novel pathogenic variant across species. The mutated amino acid is enclosed in a red rectangular box (p.Arg1265Cys). (**C**) Depiction of domains, motifs, phosphorylation sites (indicated by “P” within a circle), and pathogenic variants in BCORL1 protein. The novel pathogenic variant reported in this study is marked in red.

Karyotyping showed a normal, 44, XY complement without any chromosomal anomalies. FILTUS software [12] did not reveal any significant potentially pathogenic variants found in these regions. In addition, no potentially pathogenic autosomal recessive variants were found. Further, X-linked recessive variants were manually evaluated and revealed a novel X-linked recessive single nucleotide variant g.129159069C>T located at Xq26.1. This variant was found on exon 6 (c.3793C>T) of the *BCORL1* gene (RefSeq: NM_021946.5) which resulted in an arginine to cysteine substitution at position 1265, p.Arg1265Cys (RefSeq: NP_068765.3; CADD score: 24.4). The GRCh38 assembly of human genome reports four experimental transcripts resulting from alternative splicing and around 10 predicted transcripts for *BCORL1* gene. Exon 6 is consistently intact in all transcripts and the variant is carried in all the known transcripts. SIFT predicted the effect of this variant as 100% damaging (score: 0). Polyphen-2 predicted the effect of this variant as probably damaging with a score of 1 (sensitivity: 0 and specificity: 1). MutationTaster predicted it as disease-causing with the probability score of 1. Segregation analysis revealed hemizygous variant in affected siblings and no variant in father. The unaffected mother was the likely carrier but her carrier status could not be evaluated as she is not alive. Sanger sequencing validated the identified pathogenic variant and confirmed the segregation of the variant in both the affected individuals and the unaffected father (Figure 2A). Sequence alignment of the region of BCORL1 protein bearing the pathogenic variant across closely related species revealed a high degree of conservation (Figure 2B). PhastCons100way_vertebrate score annotated by ANNOVAR for this variant was 1 showing high conservation of the site of variant across 100 vertebrate species. This variant was not found in the 1000 genomes project. A heterozygous allele of this variant is reported in the gnomAD database in a female. This female is from the East Asian population with minor allele count of 1 in 13,861 individuals (minor allele frequency: 0.00007214). Overall gnomAD minor allele frequency was 0.000005451.

## 4. Discussion

Here, we present two male siblings exhibiting similar phenotypic characteristics of Shukla-Vernon syndrome with overlapping features of developmental delay, intellectual disability, and dysmorphic features which include broad forehead, dysmorphic ears, and long fingers. Additionally, seizures were observed in these patients as originally described in Shukla-Vernon syndrome. However, behavioral abnormalities reported in Shukla-Vernon syndrome including aggressive behavior, autism spectrum disorder, or ADHD were not observed in these patients. Additional facial dysmorphic features like bushy prominent eyebrows, synophrys, prominent beaked nose, protuberant lower jaw, and other features such as clinodactyly and kyphosis observed in these patients were not reported previously in Shukla-Vernon syndrome.

A novel, potentially pathogenic hemizygous variant in exon 6 of the *BCORL1* gene has been identified which resulted in an arginine to cysteine substitution at position 1265 in the protein product. GTEx portal, a resource for RNA-Seq based gene expression across 54 tissues showed high expression in cerebellar hemisphere (TPM:8) and cerebellum (TPM:8.3) among 13 brain tissues tested (https://www.gtexportal.org/home/gene/BCORL1, accessed on 15 February 2021). Expression Atlas showed expression of *BCORL1* in the developing brain, particularly in cerebellum (https://www.ebi.ac.uk/gxa/genes/ensg00000085185, accessed on 15 February 2021). Notably, cerebellar atrophy was observed in one patient with Shukla-Vernon syndrome [1].

The *BCORL1* gene spans ~75 kb region and one of the two transcripts, containing 13 exons, encodes a protein of 1711 amino acids (RefSeq: NP_068765.3). The protein product of *BCORL1* contains a CtBP binding domain (CBD) with a P*X*DLS motif (621–627), two tandem ankyrin repeats (ANK: 1455–1484 and ANK: 1488–1517), a putative bipartite nuclear localization signal (NLS: 1328–1336), two L*XX*LL nuclear receptor recruitment motifs (1472–1476 and 1696–1700) that are found in coregulator proteins, and a polycomb group ring finger (PCGF) Ub-like fold discriminator (PUFD) domain (1594–1711) in the C-terminus of BCORL1 (Figure 2C). Apart from these, six potential sites of phosphorylation have been reported at positions Ser496, Ser599, Ser613, Ser1029, Ser1033, and Ser1162 (UniProt accession: Q5H9F3) (Figure 2C). BCORL1 interacts with CtBP through a PXDLS motif in the CBD domain which is essential for CtBP-mediated transcription repression [3] and the PUFD domain interacts with PCGF1 and PCGF3 proteins which are components of polycomb repressive complex 1 (PRC1.1), a complex involved in gene silencing [4,5]. The previously reported disease-causing variants in BCORL1 protein are p.Pro32Leu, p.Ser496Phe, p.Val782Glu, and p.Asn820Ser, and the pathogenic variant identified in this study is p.Arg1265Cys (Figure 2C). Interestingly, these disease-causing variants are not located in any of these domains or motifs except p.Ser496Phe variant which is a potential site of phosphorylation. These variants are not located in CBD domain or in the PUFD domain that could affect the CtBP-mediated transcription repression or PUFD-mediated gene repression. The variants could create steric hindrance for the binding of CtBP, HDACs, and/or PCGF proteins. The solution structure of the PUFD domain is available but not for the complete protein. Homology modelling could not be performed because no homologous targets were found.

## 5. Conclusions

The mechanism of *BCORL1* pathogenic variants that leads to intellectual disability and other phenotypes is currently unknown. Understanding the effects of these pathogenic variants that might cause conformational changes of BCORL1 will shed light on possible alteration of binding sites of currently known complexes. Additionally, corepressor proteins can be integral components of several transcription repression complexes. Studying additional binding partners for BCORL1, particularly in cells of neurological origin or in animal model studies, can reveal these novel mechanisms.

## Figures and Tables

**Table 1:** Comparison of phenotypic and genotype details of patients (IV-2 and IV-7) with the previously published patients.

Shukla et al., 2019						Present Study	
Characteristics	Patient 1	Patient 2	Patient 3	Patient 4	Patient 5	Patient 1	Patient 2
BCORL1 variant	c.2345T>A p.(Val782Glu)	c.1487C>T p.(Ser496Phe)	c.95C>T p.(Pro32Leu)	c.95C>T p.(Pro32Leu)	c.95C>T p.(Pro32Leu)	c.3793C>T p.Arg1265Cys	c.3793C>T p.Arg1265Cys
Sex	Male	Male	Male	Male	Male	Male	Male
Gestation	32 weeks	40 weeks	40 weeks	Term	Term	Term	Term
Age at the time of examination	15 years 3 months	7 years	15 years	4 years	3 years 4 months	35 years	24 years
Weight at the time of examination	52.7 kg (−0.53 SD)	17.8 kg (−1.9 SD)	33 kg (−3.05 SD)	11 kg (−3.25 SD)	9.6 kg (−3.6 SD)	Not available	Not available
Height at the time of examination	56 cm (+0.71 SD)	53 cm (+0.74 SD)	52 cm (−1.9 SD)	47. 5 cm (−2 SD)	45 cm (−3.4 SD)	152 cm (−3.4 SD)	161 cm (−2.1 SD)
Intellectual disability	Mild	No	Severe	Severe	Severe	Moderate (IQ = 39)	Moderate (IQ = 47)
Motor delay	Mild	Mild	Yes	No	Yes	Yes	Yes
Developmental milestones	First word, 8 months Walking, 24 months	Not available	No speech attained Walking, 3 years 6 months	First word, 3 years Walking, 1 year	First word, 3 years 6 months Walking, 1 year 6 months	Walking with support, 2 years Running, 7 years Two meaningful words, >5 years Short sentences, 20 years Drinking from glass unassisted, 5 years Feeding self, 10 years Dressing self, 10 years Schooling at 4 years	Walking without support, 7 years Running, 10 years Two meaningful words, 5 years Sentence, 10 years Drinking from glass unassisted, 5 years Fully toilet-trained, 10 years Schooling at 4 years Left school
Seizures	No	No	At 1 year of age	At 2 years of age	At 6 months of age	No	Yes, at the age of 7 years
Episodes of facial weakness	No	No	No	No	No	Yes	No
Behavioural abnormalities	Autism spectrum disorder, impulsive behavior, mild aggressive behavior, ADHD	Autism spectrum disorder, impulsive behavior, aggressive behavior, attention-deficit hyperactivity disorder	Autism-spectrum disorder	Autism-spectrum Disorder	Autism-spectrum disorder	None; quiet and co-operative	None; quiet and co-operative

## Data Availability

The BCORL1 variant identified in this study has been submitted to the ClinVar database (https://www.ncbi.nlm.nih.gov/clinvar/, accessed on 15 February 2021). The accession number is SUB7745639. The data that supported the finding of this report are available upon reasonable request. The data are not publicly available due to privacy and ethical restrictions.
